# Rules-Based Real-Time Gait Event Detection Algorithm for Lower-Limb Prosthesis Users during Level-Ground and Ramp Walking

**DOI:** 10.3390/s22228888

**Published:** 2022-11-17

**Authors:** Aliaa Gouda, Jan Andrysek

**Affiliations:** 1 Institute of Biomedical Engineering, University of Toronto, Toronto, ON M5S 3G9, Canada; 2 Bloorview Research Institute, Holland Bloorview Kids Rehabilitation Hospital, Toronto, ON M4G 1R8, Canada

**Keywords:** gait event detection, gait analysis, real-time analysis, signal processing, wearable technology, ramp walking

## Abstract

Real-time gait event detection (GED) using inertial sensors is important for applications such as remote gait assessments, intelligent assistive devices including microprocessor-based prostheses or exoskeletons, and gait training systems. GED algorithms using acceleration and/or angular velocity signals achieve reasonable performance; however, most are not suited for real-time applications involving clinical populations walking in free-living environments. The aim of this study was to develop and evaluate a real-time rules-based GED algorithm with low latency and high accuracy and sensitivity across different walking states and participant groups. The algorithm was evaluated using gait data collected from seven able-bodied (AB) and seven lower-limb prosthesis user (LLPU) participants for three walking states (level-ground walking (LGW), ramp ascent (RA), ramp descent (RD)). The performance (sensitivity and temporal error) was compared to a validated motion capture system. The overall sensitivity was 98.87% for AB and 97.05% and 93.51% for LLPU intact and prosthetic sides, respectively, across all walking states (LGW, RA, RD). The overall temporal error (in milliseconds) for both FS and FO was 10 (0, 20) for AB and 10 (0, 25) and 10 (0, 20) for the LLPU intact and prosthetic sides, respectively, across all walking states. Finally, the overall error (as a percentage of gait cycle) was 0.96 (0, 1.92) for AB and 0.83 (0, 2.08) and 0.83 (0, 1.66) for the LLPU intact and prosthetic sides, respectively, across all walking states. Compared to other studies and algorithms, the herein-developed algorithm concurrently achieves high sensitivity and low temporal error with near real-time detection of gait in both typical and clinical populations walking over a variety of terrains.

## 1. Introduction

### 1.1. Overview

Gait event detection (GED), identification of key instances during a gait cycle such as foot-strike (FS) and foot-off (FO), is important for applications involving gait assessments, intelligent assistive devices such as microprocessor-based prostheses and exoskeletons, and gait training systems [1,2]. In free-living environments, GED is most commonly achieved using inertial measurement units (IMUs) which are robust, inexpensive, wearable, and compact and have minimal power requirements [3]. This makes these systems highly suitable for applications such as telerehabilitation and gait monitoring in free-living environments, to improve diagnosis, track rehabilitation progress, or assess treatment effectiveness [4,5,6]; it also makes it possible for advanced microprocessor-based assistive prosthetic and orthotic devices to function [7]. However, for accurate and reliable in-community GED, the systems must not only be capable of discerning the walking conditions (i.e., terrain, type of gait, activity), but also be robust when gait is atypical and gait deviations or anomalies are present as in the case of clinical populations with lower limb impairments including leg amputation, stroke, or others [8]. 

### 1.2. State of the Art, Problem, and Aims

A variety of state-of-the-art techniques have been used for IMU-based GED such as machine learning (ML) [9,10] and rules-based algorithms [11,12]. Previous studies have used ML-based algorithms to develop adaptable GED models suitable for specific patient populations’ gait [13,14,15]. A major downside of ML approaches is the need for training models that require large datasets that are not readily available for clinical populations [13]. Rules-based algorithms, which follow a strict logical sequence, have been used with clinical populations having gait abnormalities [11,16,17,18,19]. However, many of those algorithms are dependent on a pre-set threshold for gait events such as FS and FO [11,20]. For example, Maqbool et al. used a threshold of 20 degrees/second and a rate of change of 10 degrees/s in 80 ms for FO and FS detection, respectively. Using constant thresholds can limit the adaptability of the algorithm to different walking states (i.e., level-ground, ramp walking, etc.), as the amplitude of the inertial signals can vary between walking states [21]. In addition, the threshold values may not always be translatable between clinical populations with different gait patterns [22].

Moreover, for both ML and rules-based techniques, their implementation may be dependent on large data window sizes to achieve accurate results, and therefore, they are not well suited for real-time applications [7,23]. For example, many of the previously validated rules-based algorithms include window sizes consisting of multiple gait cycles to determine an appropriate threshold value to use for GED [20]. For example, in Aftab et al.’s study, the algorithm first identifies all of the largest positive peaks to select all of the mid-swing events before identifying other events such as FS or FO [24]. For real-time application, this significantly increases the processing time of GED. Finally, clinical populations tend to have more variable gait patterns than able-bodied (AB) individuals and together with different walking states can challenge the robustness of both rules-based and ML algorithms.

Therefore, the existing overarching problem is the need for GED algorithms that can provide accurate real-time measurement of key temporal events under a range of walking conditions and for diverse users (i.e., clinical populations) with minimal training or extensive personalization. As described above and further detailed in Section 4, to the best of our knowledge, no algorithms currently exist that concurrently satisfy all of these requirements. 

In this regard, the goal of this work was to develop and test an algorithm to accurately detect two key gait events (FS and FO) which are the basis for measuring clinically important gait parameters such as cadence, stride time, step time, and single and double support times. The algorithm was to (1) not require training datasets, (2) be robust across different walking states and (3) clinical and non-clinical populations, and (4) have minimal latency for use in real-time applications. Specifically, a rules-based algorithm was developed and validated with both AB and lower-limb prosthesis user (LLPU) participants under several walking states (i.e., level-ground walking, ramp ascent and descent). Based on previous literature (see Section 4, the Discussion section) our goal was to achieve a sensitivity of >90%, an error of <50 ms, and a latency of less than 50 ms.

## 2. Materials and Methods

### 2.1. Algorithm Design

**Figure 1:**
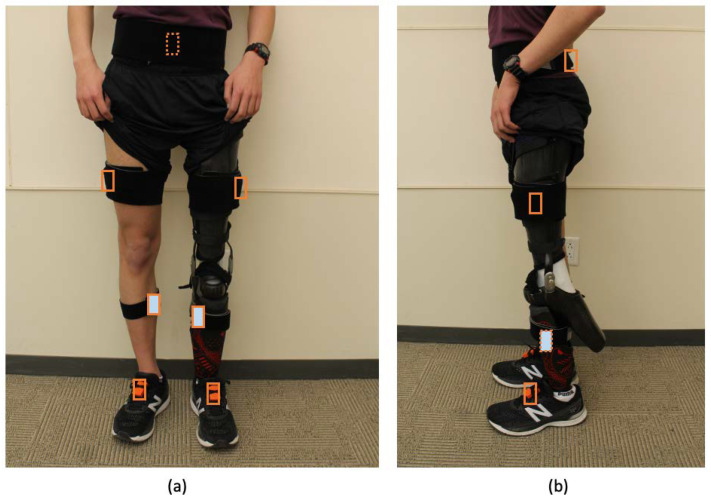
LLPU participant equipped with the Xsens MVN suit: (**a**) frontal view; (**b**) sagittal view. Sensors were attached to the feet (middle of the bridge of foot), lower leg (flat on the shin bone, medial surface of the tibia, approximately 5 cm below the patella), upper leg (middle of the lateral thigh, above the knee), and pelvis (flat on the sacrum) [32]. Solid and dashed outlines represent visible and not visible sensors, respectively. Lower-leg sensors (highlighted by filled light blue boxes) were used for validating the proposed GED algorithm.

The developed GED algorithm uses the *y*-axis (frontal–sagittal axis) raw angular velocity (gyroscope) signal from a triaxial IMU placed on the lower leg below the knee joint on each leg as in Figure 1. This location was selected as most previously reported GED algorithms used the same or similar location, due to the convenience of strapping the sensors on the lower legs [12,20,25]. The IMU signals were acquired using the Xsens MVN Awinda (Xsens North America Inc., El Segundo, CA, USA), as described in Section 2.2.

The algorithm, presented in Table 1, is based on a sequence of zero-crossings and min/max conditions [20,26]. A window of 3 samples (i − 1, i, I + 1 samples), which is 30 ms based on a sampling frequency of 100 Hz, is used. A window size of 3 allows for local minima and maxima to be detected. Compared to a previous study [20] which used a 200 ms window, this reduces the processing time significantly. The zero-crossing (ZC) state of the signal at the ith sample is always determined for each sample. The ZC state is then stored as a binary flag (i.e., 0, 1). The first mid-swing (MSW) is detected as the first minimum greater than abs(2) rad/s after descending ZC. This threshold was used for MSW since it can be easily translated between participants and populations, as every participant exhibited a MSW value much lower than −2 rad/s from the data collected. Additionally, based on previous literature, MSW always occurs beyond 2 rad/s (~115 deg/s) [11,20]. FS is determined as the first maximum after the first ascending ZC following MSW detection. FO is determined as the last maximum before descending ZC. Since multiple ZCs may occur between FS and FO, a wait time of 20 samples (200 ms) is placed after FS detection. The average gait cycle from this study for AB and LLPU participants was 1.04 s and 1.2 s, respectively. Since the average stance time is 60% of the gait cycle (approximately 624 ms for AB, 720 ms for LLPU), a 200 ms delay would be well below the timing that would miss an FO or FS gait event [27]. To ensure the correct maximum is selected, all local maxima are stored, and once the descending ZC occurs, the last stored value is then selected as FO. Signals are filtered using a third-order median filter, defined as a nonlinear filter in which each output sample is computed as the median value of the input samples under the window. This avoids the use of other filters such as Butterworth filters that are commonly used in GED work but require a phase shift correction to allow for real-time detection [28]. 

### 2.2. System Instrumentation

The performance of the developed algorithm was validated by comparing the gait events (FS, FO) detected by the developed algorithm and the results from a validated reference motion capture system, the Xsens MVN Awinda. Seven inertial sensors (Xsens North America Inc., CA, USA) were attached to the feet (x2), lower leg (x2), upper leg (x2), and pelvis (x1) (Figure 1). Foot contact events were processed and extracted using the MVN Analyze software (Xsens North America Inc., CA, USA), which uses all seven sensors to reliably and accurately measure the FS and FO gait events across a wide range of gait conditions [29,30,31]. However, it is noted here that our GED algorithm uses only the raw angular velocity (gyroscope) signals from two lower-leg inertial sensors. All sensor data were sampled at a frequency of 100 Hz. 

### 2.3. Participants

Participants were recruited if they were fourteen years or older and community ambulators able to walk on level ground and ramps without ambulatory aids (except for current prosthetic device for LLPU participants). LLPU participants with unilateral amputations at any level (Symes, below-knee, above-knee) were included. Bilateral lower-limb amputations and/or participants using additional walking aids (i.e., crutches, canes, etc.) were excluded. All participants had no previously known neurological disorders.

All participants were recruited through Holland Bloorview Kids Rehabilitation Hospital. AB participants were recruited through posted recruitment bulletins, and potential LLPU participants were referred to our research group through the Prosthetics and Orthotics department at the hospital, based on the aforementioned criteria. 

Seven AB (4 females; 26.1 ± 3.4 years; height 169.5 ± 5.1 cm; weight 67.6 ± 12.8 kg) and 7 unilateral LLPU (5 males; age 27.6 ± 8.7 years; height 170.1 ± 8.7 cm; weight 63.7 ± 9.5 kg; 4 transfemoral and 3 transtibial; years since amputation median 18 (IQR 6.75, 22); years with their current prosthesis median 2 (IQR 1.15, 6.5)) participants were recruited for this study. 

### 2.4. Experimental Protocol

**Figure 2:**
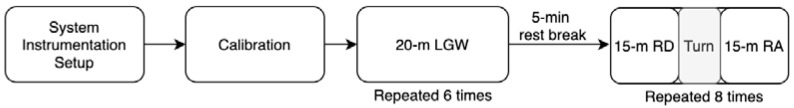
Experimental protocol overview for the data collection session. LGW: level-ground walking, RA: ramp ascent, RD: ramp descent.

Participants were involved in a single 2 h data collection session. Participants were equipped with the Xsens wearable motion capture system as outlined in Figure 1. Then, an N-pose calibration was performed for the Xsens Awinda system to initialize the orientation of the inertial sensors [25]. During data collection, subjects walked in three different walking states: level-ground walking (LGW), ramp ascent (RA), and ramp descent (RD). As outlined in Figure 2, each participant completed six 20 m straight LGW and eight 15 m RD–turn–RA walk trials. Five-minute rest breaks were taken between LGW and RA/RD. After each walking pass, data collection was stopped before the participant turned around and then restarted after the turn was completed. This was done to ensure only straight walking was included in the analysis. The experimental protocol was approved (REB-0102) by the Research Ethics Board at Holland Bloorview Kids Rehabilitation Hospital, Canada. Informed written consent from each participant was obtained before the study was conducted.

### 2.5. Data Analysis 

The developed algorithm was implemented, tested, and analyzed using a custom Python script. The script ran through each gait trial and using the *y*-axis angular velocity signal of each limb, the GED algorithm yielded all MSW, FS, and FO events. Foot contact data from the validated algorithms within Xsens were processed and exported to XML format using Xsens MVN Analyze software (Xsens North America Inc., El Segundo, CA, USA). Data were then parsed using another custom python script, which extracted all the FS and FO events from each trial. In total, 1260 steps were detected and evaluated for each side (30 steps per activity per participant). Sensitivity (Equation (1)) and temporal error (Equations (2) and (4)) were evaluated by comparing the gait events detected by the Xsens MVN Analyze software and by the developed GED algorithm respectively [33,34,35].

(1) $$\mathrm{Sensitivity}=\frac{\mathrm{True}\;\mathrm{Positives}}{\mathrm{True}\;\mathrm{Positives}+\mathrm{False}\;\mathrm{Negatives}}$$

Error (ms) = Timestamp_xsens_ − Timestamp_algorithm_ (2)

Gait Cycle Duration (ms) = FS_i_ − FS_i−1_ (3)

(4) $$\mathrm{Error}\left(\%\;\mathrm{of}\;\mathrm{Gait}\;\mathrm{Cycle}\right)=\frac{\mathrm{Error}}{\mathrm{Gait}\;\mathrm{Cycle}\;\mathrm{Duration}}\times 100\%$$

Significant differences in temporal error levels between groups (AB vs. LLPU) were statistically analyzed in JMP Pro 16 software (Statistical Discovery, SAS, Cary, NC, USA). To assess the normality of the data distribution, Shapiro–Wilk test was used (*p* < 0.05), which indicated all data to be non-normally distributed. Thus, a non-parametric two-tailed Wilcoxon signed-rank test was used to assess the difference in accuracy and sensitivity levels. Using a Bonferroni correction with an adjusted critical alpha value of 0.00417 (*p* = 0.05/12), statistical significance was adjusted to account for any potential type I errors. Since the temporal error data were found to be non-normally distributed, median and interquartile ranges were reported [36]. Finally, to assess the agreement between the results from the developed GED algorithm and the Xsens MVN Analyze software, Kendall’s W (coefficient of concordance) was calculated, since the data were found to be non-normally distributed [37]. 

## 3. Results

**Figure 3:**
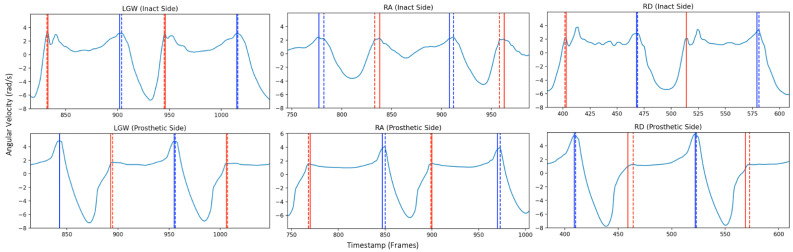
Sample data displaying GED events detected (FS and FO) for LGW, RA, and RD of both intact and prosthetic sides of LLPU_P02 (transfemoral). Plot of raw angular velocity signal (light blue). FS (blue) and FO (red), Xsens (solid) and proposed algorithm (dashed).

The overall sensitivity was 98.87% for AB and 97.05% and 93.51% for LLPU intact and prosthetic sides, respectively, across all walking states (LGW, RA, RD) (Table 2). The overall temporal error (ms) between the proposed and referenced detection for both FS and FO was 10 (0, 20) ms for AB and 10 (0, 25) ms and 10 (0, 20) ms for the LLPU intact and prosthetic sides, respectively, across all walking states (Figure 3, Table 3). The overall temporal error, as a percentage of gait cycle, was 0.96 (0, 1.92)% for AB and 0.83 (0, 2.08)% and 0.83 (0, 1.66)% for the LLPU intact and prosthetic sides, respectively, across all walking states (Table 4). The overall ICC was found to be 0.99 for both AB and LLPU groups.

When evaluating the results separately across different walking states and groups, FS and FO detection of the AB group had the highest sensitivity during all walking states, whereas for the LLPU group, sensitivity was approximately 8% lower for RA and RD walking states. On the other hand, the accuracy of FS and FO had similar ranges between each of the groups. However, accuracy decreased during RA and RD for both AB and LLPU groups. The pairwise statistical comparison results, as highlighted in Table 3, indicate a significant difference between the LLPU and AB groups for all comparisons except for FS during RA of the intact side and FS and FO during LGW and RA of the prosthetic side.

## 4. Discussion

The study presents a new rules-based algorithm that can be used in real time for FS and FO detection using a single gyroscope signal on each leg that achieved an overall mean sensitivity of >93% and a median error of <30 ms and <1% of a gait cycle for both AB and LLPU groups across three different walking states (LGW, RA, RD). Furthermore, a very high agreement (Kendall’s W = 0.99) between the developed algorithm and the Xsens results was found for both groups. 

Sensitivity and error were compared to previous studies (Table 5). However, few studies used the same methods (i.e., type of participant group, walking states, real-time application), and thus a direct comparison was not possible. Table 4 suggests that this study’s algorithm performed comparably to or better than previous studies. Most of the mentioned studies did not report sensitivity results. However, Catalfamo et al. reported an overall 99.5% sensitivity for LGW, RA, and RD when tested with an AB group [20]. The sensitivity in our study was slightly lower in all groups (98.87%, 97.05%, and 93.51% for AB, LLPU intact, and LLPU prosthetic, respectively). However, this could be due to the larger sample size in our study resulting in a higher probability of detecting outliers resulting in false negatives, which might have been missed in smaller sample size studies. 

Compared to the reported studies in Table 5, the accuracy of our algorithm achieved similar results. However, in some instances such as FO detection, the algorithm yields considerably higher accuracy results. For example, compared to the results of Maqbool et al. during RD walking, the temporal error is improved by approximately 100 ms for the LLPU group [11]. This highlights the improved accuracy exhibited by the proposed algorithm, as FO is typically more challenging to detect due to the increased variations of the gait signal prior to mid-swing [38]. The improved accuracy can be due to the proposed algorithm being independent of a constant threshold, which has been used in previous studies, when detecting FO. Therefore, any variability in the magnitude of the angular velocity signal typically observed due to the walking state (i.e., ramp ascent or descent) [39] or prosthesis technology used (i.e., hydraulic, bionic prosthesis, etc.) would not affect the accuracy [40]. As aforementioned, the overall error as a percentage of the gait cycle was <1%, with the highest median error (percentage of gait cycle) being 2.8 (1.86, 3.73)% for FS detection of the AB group during RD (Table 4). For real-time gait analysis, such an accuracy level is acceptable since it is below the coefficient of variation of AB gait, as reported by Beauchet et al. to be 4% and 3% for swing and stance time, respectively [41]. 

Although the algorithm performed better for the AB relative to the LLPU group, based on the statistical pairwise comparison, the differences were small and the LLPU group results were still comparable to other studies that evaluated their GED algorithms with the LLPU population [12,19]. This reduced performance in the LLPU (compared to AB) could be due to the variety of gait styles exhibited by the LLPU participants based on the level of their amputation and/or the type of prosthetic device used. In addition, to account for such differences, future work should investigate adaptable delays. For example, the 200 ms delay applied after FS detection can be adjusted based on the individual’s own stance time (i.e., faster/slower walking). This will ensure that in cases where an individual may be walking faster (stance time less than approximately 600 ms), the delay remains below the individual’s average stance time. 

Finally, one of the unique features of the proposed algorithm is the minimal window size required for accurate GED. The algorithm requires three samples or 30 ms of data at a time, whereas other studies have either used the overall signal to identify all the peaks at once [12,24] or used larger window sizes of 200 ms, which can be approximately 20% of an average gait cycle [20]; neither of these algorithms is suitable for real-time implementations such as biofeedback systems used for gait training and prosthetic control [19,42,43]. Such systems require a minimal delay to allow for the sensory feedback to activate. However, it is important to note that with a lower sampling frequency, the window size would increase, as it is based on the number of samples as opposed to the timing length of the window. Future work should evaluate the algorithm using different sampling rates, and with a faster sampling rate, it may be possible to reduce the latency further.

Some of the limitations of this study include the large error variability, which appears to be a consistent issue with many of the previously studied GED algorithms [11,12,19]. This could be addressed by first evaluating the GED algorithms with a significantly higher sampling rate and then identifying additional signal features that increase accuracy. Additionally, this study did not evaluate the differences between both sides of the AB group. Some studies have suggested that, although gait in healthy populations is often symmetrical with less variability, there are cases of gait asymmetry with higher variability that may occur due to factors such as fatigue or walking speed [39,44]. Thus, future studies should account for some of these factors to assess the difference in GED performance between both sides. Finally, future work should focus on improving and validating the proposed algorithm for other walking states (i.e., uneven terrain, running, stair walking) and with other patient populations (i.e., cerebral palsy, Parkinson’s). Moreover, future studies should assess the algorithm performance over longer periods of time (i.e., multiple sessions for the same participant) to evaluate the test–retest reliability. 

## 5. Conclusions

The results of this study support the use of angular velocity signals acquired from the lower legs for real-time GED in the AB and LLPU populations for gait on a variety of terrains, while avoiding the use of pre-set thresholds for FS and FO detection. The new algorithm achieved near real-time gait event detection, with error and sensitivity levels of <30 ms and >93%, respectively, using only a single sensor (signal) for each limb, which is comparable and in some cases an improvement compared to previous studies. The findings of the proposed algorithm support the translation of this work into clinical practice using low-cost gyroscopes for gait analysis, as it was tested with two distinct groups (AB and LLPU). Future work should focus on assessing the robustness of the GED algorithm for different walking states and clinical populations. Furthermore, to improve the variability of accuracy levels, future work should assess the performance for significantly higher sampling rates. 

## Figures and Tables

**Table 1:** Summary of heuristic rules for GED algorithm.

Gait Event	Conditions
Mid-swing (MSW)	MSW is based on: ▪ Find descending (positive-to-negative signal) zero crossing. ▪ Search for minimum value: ▪ **If** minimum value is <−2 rad/s, **then** save current value (timeframe) as MSW. ▪ **Else** continue minimum value search.
Foot Strike (FS)	FS is based on: ▪ MSW detection: ▪ **If** MSW for this cycle has been detected, **then** proceed. ▪ **Else** return to MSW detection search. ▪ Find ascending (negative-to-positive signal) zero crossing. ▪ Search for maximum value: ▪ **If** maximum value found, **then** save current value (timeframe) as FS. Trigger a 200 ms time delay for FO detection. ▪ **Else** return to MSW detection search.
Foot Off (FO)	FO is based on: ▪ MSW and FS detection: ▪ **If** MSW and FS for this cycle has been detected, **then** proceed. ▪ **Else** return to MSW detection search. ▪ Check 200 ms time delay is complete. ▪ Search for and store all maximum values. ▪ Find the next descending (positive-to-negative signal) zero crossing: ▪ **If** count (maximum values found) > 1, **then** save the latest value as FO. ▪ **Else** return to MSW detection search.

**Table 2:** Overall sensitivity (mean (standard deviation)) results of GED algorithm.

Walking State	Event	Sensitivity (%)		
		AB	LLPU	
		Both	Intact	Prosthetic
LGW	FS	100.00 (0.0)	98.44 (3.74)	97.64 (3.04)
	FO	99.77 (0.38)	99.8 (0.52)	98.57 (1.39)
RA	FS	98.78 (1.30)	94.52 (7.38)	89.93 (7.05)
	FO	98.08 (2.21)	95.25 (9.22)	90.44 (7.13)
RD	FS	98.28 (3.49)	98.00 (3.40)	89.93 (7.05)
	FO	98.31 (3.41)	96.31 (4.34)	90.44 (7.13)
Overall		98.87 (2.25)	97.05 (5.52)	93.51 (9.92)

**Table 3:** Temporal error (median (IQR1, IQR3)) results of GED algorithm between the proposed algorithm and validated Xsens results. Pairwise comparison (*p*-values) results between accuracy of AB and LLPU group for each mode and side.

		Error (ms)			Pairwise Comparison	
Walking State	Event	AB	LLPU		AB—Intact	AB—Prosthetic
		Both	Intact	Prosthetic		
LGW	FS	10 (0, 20)	10 (0, 30)	10 (0, 20)	**<0.001**	0.779
	FO	10 (0, 20)	10 (0, 20)	10 (0, 30)	**<0.001**	0.407
RA	FS	−10 (−20, 0)	−20 (−30, −10)	−20 (−30, 0)	0.319	0.322
	FO	20 (10, 30)	20 (20, 30)	20 (10, 30)	**<0.001**	0.006
RD	FS	30 (20, 40)	20 (20, 30)	10 (0, 20)	**00.004**	**<0.001**
	FO	−10 (−20, 10)	0 (−10, 10)	10 (0, 20)	**<0.001**	**<0.001**
Overall		10 (0, 20)	10 (0, 25)	10 (0, 20)	-	-

Negative and positive values indicate that the proposed algorithm leads or lags the reference Xsens results, respectively. Statistically significant differences are highlighted in **bold** font (adjusted critical alpha value = 0.05/12 = 0.0041667).

**Table 4:** Temporal error (median (IQR1, IQR3)) and corresponding gait cycle length (mean (standard deviation)) results of GED algorithm.

		Error (% of Gait Cycle)			Gait Cycle Length (s)		
Walking State	Event	AB	LLPU		AB	LLPU	
		Both	Intact	Prosthetic	Both	Intact	Prosthetic
LGW	FS	0.96 (0, 1.92)	0.85 (0, 2.55)	0.85 (0, 1.69)	1.04 (0.06)	1.18 (0.14)	1.18 (0.14)
	FO	0.93 (0, 1.86)	0.78 (0, 1.56)	0.78 (0, 2.35)			
RA	FS	−1 (−2, 0)	−1.74 (−2.61, −0.87)	−1.73 (−2.6, 0)	1.07 (0.13)	1.28 (0.17)	1.28 (0.16)
	FO	1.92 (0.96, 2.88)	1.7 (1.7, 2.55)	1.69 (0.85, 2.54)			
RD	FS	2.8 (1.86, 3.73)	1.56 (1.56, 2.34)	0.78 (0, 1.57)	1.00 (0.07)	1.15 (0.15)	1.15 (0.16)
	FO	−1 (−2, 1)	0 (−0.87, 0.87)	0.87 (0, 1.73)			
Overall		0.96 (0, 1.92)	0.83 (0, 2.08)	0.83 (0, 1.66)	1.04 (0.09)	1.20 (0.15)	1.20 (0.16)

**Table 5:** Summary of results of previous studies with at least partially comparable results [11,12,19,20,24,26].

Study	Participant Population	Activity	Detection Method	FS Error (ms)	FO Error (ms)	Real-Time Analysis
Aftab et al. [24]	LLPU (n = 10)	LGW	Shank velocity derived from marker data	Mean: −8 (prosthetic) 1 (intact)	Mean: 35 (prosthetic) 84 (intact)	No
Zahradka et al. [26]	AB (n = 11), CP (n = 6)	Treadmill LGW	Shank angular velocity algorithm	−33.41 ± 0.86	−56.20 ± 1.02	No
Catalfamo et al. [20]	AB (n = 7)	LGW	Shank angular velocity algorithm: threshold- and ZC-based	[−16, 1]	[37, 63]	Window size = 200 ms
		RA		[−35, −8]	[34, 52]	
		RD		[−29, 12]	[60, 85]	
Simonetti et al. [19]	LLPU (n = 7)	LGW	Shank mediolateral angular velocity, flexion–extension angle, and axial acceleration	Mean: −30 (prosthetic) −10 (intact)	Mean: −10 (prosthetic) −50 (intact)	No
Maqbool et al. [12]	AB (n = 4) LLPU (n = 1)	LGW	Shank angular velocity (sagittal) and linear acceleration (longitudinal) algorithm; threshold-based only	17 ± 11.4 (AB) 21.8 ± 20 (LLPU, prosthetic) 12 ± 9.5 (LLPU, intact)	−15.5 ± 22 (AB) −7.5 ± 15.5 (LLPU, prosthetic) −23.8 ± 8 (LLPU, intact)	Window size not reported
Maqbool et al. [11]	AB (n = 8) LLPU (n = 1)	RA	Shank angular velocity: threshold-based only	[11, 17] (AB) [14, 49] (LLPU, prosthetic) [8, 18] (LLPU, intact)	[−10, 0.2] (AB) [−39, 27] (LLPU, prosthetic) [−43, −16] (LLPU, intact)	Window size not reported
		RD		[10.5, 17] (AB) [−19, 19] (LLPU, prosthetic) [−0.3, 17] (LLPU, intact)	[−25, 36] (AB) [−141, 105] (LLPU, prosthetic) [−44, −26] (LLPU, intact)	

AB: able-bodied; LLPU: lower-limb prosthesis user; CP: cerebral palsy; LGW: level-ground walking; RA: ramp ascent; RD: ramp descent; ZC: zero-crossing.
